# Sex differences in the association of planetary health diet, genetic risk, with overall cancer incidence: a prospective study from UK Biobank

**DOI:** 10.1097/JS9.0000000000002711

**Published:** 2025-06-24

**Authors:** Yue Han, Jia-Cheng Liu, Ying-Ying Zhang, Yu Li, Xi Chen, Bang-Quan Liu, Dong-Run Li, He-Li Xu, Wen-Rui Zheng, Fang-Hua Liu, Yi-Zi Li, Yi-Fan Wei, Fan Cao, Qi-Jun Wu, Fu-Lan Hu, Ting-Ting Gong

**Affiliations:** a Department of Obstetrics and Gynecology, Shengjing Hospital of China Medical University, Shenyang, China; b Department of Clinical Epidemiology, Shengjing Hospital of China Medical University, Shenyang, China; c Key Laboratory of Precision Medical Research on Major Chronic Disease, Shengjing Hospital of China Medical University, Shenyang, China; d Department of Epidemiology, School of Public Health, China Medical University, Shenyang, China; e Department of Biostatistics and Epidemiology, School of Public Health, Shenzhen University Health Science Center, Shenzhen, China; f Department of Epidemiology, School of Public Health, Harbin Medical University, Harbin, China; g NHC Key Laboratory of Advanced Reproductive Medicine and Fertility (China Medical University), National Health Commission, Shenyang, China

**Keywords:** genetic risk, overall cancer, planetary health diet, prospective cohort study

## Abstract

**Background:**

The effect of planetary health diet (PHD) may differ by sex, and the associations with cancer risk remain unclear. This study aimed to investigate sex differences in the associations between PHD and overall cancer risk, as well as the joint effects of PHD and genetic risk.

**Methods:**

This study included 177 441 participants from the UK Biobank. The PHD score was calculated by summing the scores of 14 dietary components, each assessed on an adjustable 0–10 scoring system. To evaluate genetic risk, an overall cancer polygenic risk score (CPRS) was constructed using 1025 single-nucleotide polymorphisms. The outcome was overall cancer, defined by 20 site-specific cancers.

**Results:**

During a median follow-up of 12.98 years, 15 476 cancer cases were identified, comprising 82 146 men and 95 295 women. In the multivariable-adjusted model, participants in the highest quintile of PHD adherence showed a borderline significant reduction in overall cancer risk (hazard ratio [HR]: 0.95, 95% confidence intervals [CI]: 0.90–1.00) relative to the lowest quintile. Additionally, each standard deviation increase in PHD score was associated with a 4% reduction in overall cancer risk (HR: 0.96, 95% CI: 0.95–0.98). When stratified by sex, individuals with the highest PHD score were associated with lower cancer risk in men (HR: 0.92, 95% CI: 0.86–0.99), but not in women (HR: 0.96, 95% CI: 0.89–1.03). In the joint analysis, individuals with high PHD scores and low CPRS had the relatively lowest risk of overall cancer compared to those with low PHD scores and high CPRS, in both men (HR: 0.39, 95% CI: 0.33–0.46) and women (HR: 0.55, 95% CI: 0.47–0.65).

**Conclusion:**

The PHD was associated with a reduced overall cancer risk among men. Individuals with high PHD scores and low CPRS had the relatively lowest cancer risk. These findings highlight that the PHD may be particularly beneficial in men for cancer prevention.

**HIGHLIGHTS**

- The first cohort study to assess links between planetary health diet (PHD), genetic, and cancer risk by sex.
- The PHD was associated with a reduced overall cancer risk in men.
- Individuals with high PHD scores and low cancer polygenic risk score had lowest cancer risk.
- These findings suggest that the PHD may be beneficial in cancer prevention in men.

## Introduction

Cancer has become a critical global health issue, ranking among the leading causes of death and disease burden worldwide. In 2022, global cancer statistics reported nearly 20 million new cases and approximately 10 million cancer-related deaths[1]. Notably, cancer incidence is significantly higher in men compared with women, with disparities reaching four to five times in some regions[1]. Due to an aging population, increasing unhealthy lifestyles, and environmental exposures, the global cancer incidence is projected to reach nearly 28 million new cases annually by 2040[2]. The rise in cancer incidence among men is expected to surpass that in women, underscoring the need for gender-specific prevention and treatment strategies.

Cancer development is shaped by a combination of genetic factors and lifestyle habits, such as smoking, alcohol intake, and diet^[3,4]^. Under certain conditions, the disease burden linked to diet surpasses that caused by tobacco and alcohol[5]. The planetary health diet (PHD), introduced by the EAT-Lancet Commission in 2019, promotes a dietary pattern that seeks to balance human health with the planet’s sustainable development[6]. This dietary pattern emphasizes diversity by encouraging greater consumption of plant-based foods like vegetables, fruits, whole grains, and legumes, while limiting red meat, processed meats, and refined sugars. Recent studies have indicated that following the PHD is associated with protective benefits against cardiovascular disease and lower all-cause mortality, with some variations observed between sexes^[7–9]^. However, evidence linking the PHD to cancer risk shows certain sex differences but remains limited and inconsistent. Findings from the NutriNet-Santé cohort suggested that the association between the PHD and overall cancer risk was observed only in women, while no significant association was observed in the general population[10]. In contrast, a large prospective cohort study highlighted significant sex-specific differences, with a reduced risk of cancer associated with stronger adherence to the PHD observed only in men[11]. Although the biological mechanisms underlying the observed sex differences in the association between PHD adherence and cancer risk remain to be fully elucidated, previous research has suggested that sex-specific factors, such as differential gene expression[12] and hormone-related immune responses[13], may contribute to the varying impacts of lifestyle factors on cancer risk between men and women. Therefore, further research is needed to better understand the relationship between PHD adherence and cancer risk, particularly focusing on the potential modifying role of sex.

The genetic background of an individual contributes to the development of specific cancers, such as prostate and colorectal cancer^[14,15]^. Previous research has indicated that polygenic risk scores (PRS), generated through genome-wide association studies (GWAS) by integrating multiple genetic loci to construct cancer polygenic risk scores (CPRS), are effective in predicting an individual’s susceptibility to certain genetically driven cancers^[16,17]^. The interaction between dietary patterns and genetic risk collectively influences both the occurrence and progression of cancer^[18–20]^. For example, following a healthy diet rich in fiber and low in fat can reduce the 5-year incidence risk of upper gastrointestinal cancer in individuals carrying moderate-to-high genetic risk levels[21]. Individuals with greater adherence to a plant-based diet and lower genetic risk demonstrate the lowest risk of colorectal cancer, with the association showed sex differences[22]. These findings suggest that sex differences might have an impact on the associations among the PHD, genetic risk, and cancer incidence.

This study utilized the UK Biobank (UKB) database to investigate the associations between the PHD, genetic risk, and overall cancer risk, with a focus on the sex differences. Additionally, this study also aimed to assess the combined effects of PHD and genetic risk on overall cancer risk.

## Methods

### Study population

Data were obtained from the UKB, with the study design comprehensively described in previous publications[23]. Briefly, the UKB is a large prospective cohort study aimed at investigating how genetic and lifestyle factors influence disease. A total of 502 411 participants aged 37–73 were recruited from 22 centers across England, Scotland, and Wales between 2006 and 2010. Participants completed a touchscreen questionnaire, participated in a brief verbal interview, underwent physical measurements, and provided biological samples. The UKB study received approval from the North West Multicenter Research Ethical Committee (reference number 21/NW/0157), and all participants provided written informed consent at enrollment.

**Figure 1.**
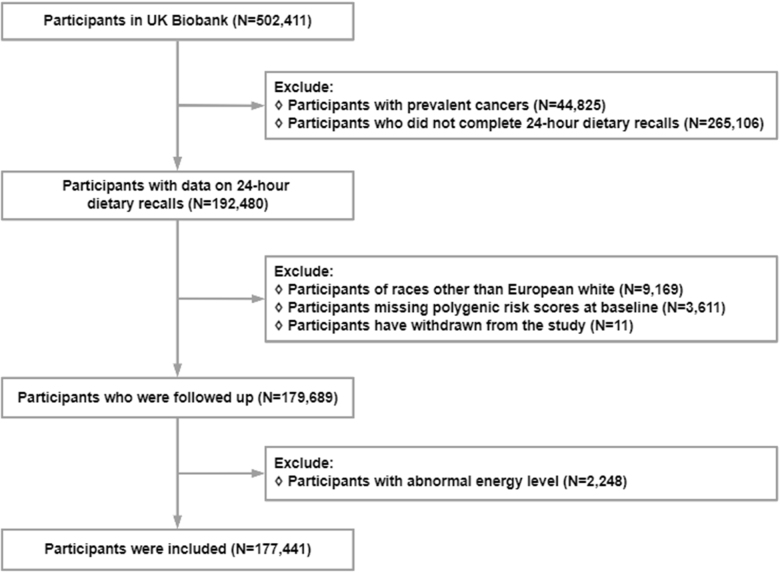
Flowchart for the selection of participants from the UK Biobank.

In the study (application number 81680), we excluded individuals with cancer at baseline (*n* = 44 825), those who failed to complete the dietary questionnaire (*n* = 265 106), those who were not classified as white individuals of European ancestry (*n* = 9169), those lacking data for constructing PRS at baseline (*n* = 3611), those who dropped out or were lost to follow-up from the UKB (*n* = 11), and those with implausible energy intake (men: <800 or >4000 kcal/day; women: <500 or >3500 kcal/day; *n* = 2248)[24]. Ultimately, 177 441 participants were included in the analysis (Fig. 1). The cohort study has been reported in line with STROCSS 2025 criteria[25].

### Dietary assessment

The UKB employed a validated online tool for 24-hour dietary assessments known as Oxford WebQ to collect comprehensive data on the quantity and types of food consumed^[26,27]^. The Oxford WebQ tool, an online dietary assessment instrument, included questions regarding the consumption of 206 commonly consumed foods and 32 types of beverages over the previous 24 hours. It has been confirmed for accuracy through comparison with an interviewer-administered 24-hour dietary recall[28] and biomarkers[29], demonstrating satisfactory consistency across two dietary assessments[26]. The initial dietary assessment took place at the assessment centers between April 2009 and September 2010, followed by four additional online questionnaires after recruitment ended. Invitations were sent via email every 3–4 months from February 2011 to June 2012. For participants who completed the assessment multiple times, the average intake of each food item was calculated across all dietary assessments.

### PHD score definition

Based on the intake recommendations from the EAT-Lancet Commission, the PHD score was established (Supplemental Digital Content Table 1, available at: http://links.lww.com/JS9/E414). The evaluation standards are outlined in prior publications[7] and provided in Supplemental Digital Content Table 2, available at: http://links.lww.com/JS9/E414. Specifically, fourteen dietary components were included in the PHD, classified into three groups depending on their associations with health outcomes[30]. In the current study, adequacy components consist of vegetables, fruits, nuts, fish, legumes, and unsaturated fats, optimum components include whole grains, dairy products, eggs, potatoes, and poultry, moderation components comprise saturated fats, red meat, and added sugars. Each component was evaluated based on a 2500 kcal/day intake[31], with scores ranging from 0 (non-adherence) to 10 (highest adherence). Adequacy components use a linear scale where higher intake directly increases the score, with 10 points awarded for meeting or exceeding the recommended maximum. Moderation components follow an inverse scale, rewarding lower consumption (10 points for minimal intake) and penalizing excess (0 point for exceeding limits). Balanced components require strict adherence to target intake levels: a perfect score (10 points) is awarded only at the exact recommendation, while deviations, whether below or above, resulted in proportional deductions (e.g. 50% or 150% of the target intake reduces the score by half)[31].

### PRS calculation and CPRS construction

For cancer types, we systematically searched GWAS of cancer in populations of European ancestry published before January 1, 2020, in PubMed, supplemented by a search of the NHGRI-EBI GWAS Catalog[32]. We then estimated site-specific PRS based on SNPs for overall cancer. A PRS for each cancer site was constructed using an additive model, as previously described[16]. Briefly, the dosage of each individual’s risk allele was multiplied by its corresponding effect size for the specific cancer site and then summed. Specific details and construction methods are provided in the Supplemental Digital Content Methods, available at: http://links.lww.com/JS9/E414.

To better assess genetic susceptibility to cancer, we used a CPRS, a widely adopted approach for capturing the cumulative effects of multiple genetic variants on overall cancer risk^[19,33]^. The CPRS was built to serve as an indicator of genetic risk for overall cancer, following this process:

$$CPRS_{i}=\sum_{k=1}^{k}h_{k}PRS_{i,k}$$

The CPRS for the *i*th individual is denoted as PRS*_i_*_,*k*_, where *h_k_* represents the incidence of cancer type *k* in the UK population (Supplemental Digital Content Methods, available at: http://links.lww.com/JS9/E414). To ensure a consistent and comparable estimation of genetic risk, age-standardized incidence rates for cancer were used[34]. Based on their CPRS, participants were categorized into three genetic risk groups: low risk (lowest quintile), intermediate risk (quintiles 2–4), and high risk (highest quintile)[19]. This stratification allowed the identification of individuals at extreme genetic risk and increases the observed effect size of CPRS.

### Outcome assessment

Our primary outcome of interest was cancer incidence in the population. Data on diagnosis dates and cancer types were obtained through linkage to population-based cancer registries, including the National Cancer Data Repository, the Scottish Cancer Registry, and the Welsh Cancer Surveillance & Intelligence Unit[35]. The follow-up period ended on May 31, 2022, or at the time of cancer diagnosis, whichever occurred first. Cancer diagnoses were verified using the 10th revision of the International Classification of Diseases[36].

### Assessment of covariates

Sociodemographic details (age at study entry, sex, education level, and annual household income), lifestyle factors (energy intake, alcohol consumption, smoking status, Body Mass Index [BMI], and Townsend deprivation index), along with family history of cancer, were collected via a touchscreen questionnaire at the baseline assessment centers. Further details about the covariates are provided in the Supplemental Digital Content Methods, available at: http://links.lww.com/JS9/E414.

### Statistical analyses

Baseline characteristics were expressed as means (standard deviation [SD]) or medians (interquartile range [IQR]) for continuous variables, and as counts (percentages) for categorical variables, categorized by PHD score quintiles. Continuous variables were analyzed using the Student’s *t*-test or Kruskal–Wallis test, while categorical variables were evaluated with the Chi-square test.

Hazard ratios (HRs) and 95% confidence intervals (CIs) for the associations between PHD score, CPRS, and cancer risk were estimated using Cox proportional hazards regression models. The proportional hazards assumption was verified using the Schoenfeld residual method (*P* > 0.05). The PHD score was analyzed by quintiles (lowest quintile as reference) and as a continuous variable (per 1-SD) to assess associations with cancer incidence in the overall population and by gender. Restricted cubic spline (RCS) models were applied to explore the nonlinear relationship between PHD scores and cancer risk. CPRS was also evaluated as a categorical variable (low group as reference) and as a continuous variable (per 1-SD) for men and women. Based on previous studies, different variables were adjusted in the model[31]. The crude model had no covariate adjustments. Model 1 adjusted for age, sex, BMI, total energy intake, and the first ten genetic principal components. Model 2 included additional adjustments for income, education, Townsend deprivation index, alcohol consumption, smoking status, and family history of cancer.

To assess the joint associations of PHD and genetic susceptibility with the cancer incidence rate, we classified males and females into 15 groups separately based on PHD scores (Q1–Q5 by quintiles) and CPRS (low, intermediate, and high). The HRs of cancer incidence rates in various groups were estimated compared to those with high CPRS as well as the Q1 PHD score.

To examine if the associations between PHD score and overall cancer risk differed among subgroups, we conducted analyses stratified by BMI (<24, 24–28, >28 kg/m^2^), age (<60, ≥60 years), smoking status (never, previous, current), and alcohol consumption (never, previous, current). Multiple sensitivity analyses were performed to evaluate the stability of the results. First, individuals who developed cancer within first 2 years of completing the last 24-hour dietary recall were excluded to reduce reverse causality. Next, participants who did not complete the questionnaire or completed it only once were excluded. Finally, the primary analyses were repeated by reconstructing the PHD score using the method developed by Knuppel *et al*[37].

Statistical analyses were conducted with R software (version 4.3.1), with a two-sided *P*-value below 0.05 deemed significant.

## Result

### Participants and characteristics

In this study, we included 177 441 participants in the final analysis, comprising 82 146 men and 95 295 women. During a median follow-up of 12.98 years (IQR: 12.34–13.80 years), 15 476 new cancer cases (8.72%) were recorded, including 8307 (10.11%) in men and 7169 (7.52%) in women. Baseline characteristics classified by the quintiles of the PHD score are presented in Table 1 and Supplemental Digital Content Tables 3 and 4, available at: http://links.lww.com/JS9/E414.

**Table 1:** Baseline characteristics of included participants according to the planetary health diet score

Characteristics	Total (*N* = 177 441)	Quintiles of planetary health diet score					*P*-value
		*Q*1 (*N* = 35 489)	*Q*2 (*N* = 35 488)	*Q*3 (*N* = 35 488)	*Q*4 (*N* = 35 488)	*Q*5 (*N* = 35 488)	
Cancer incidence	15 476 (8.72)	3127 (8.81)	3129 (8.82)	3049 (8.59)	3115 (8.78)	3056 (8.61)	0.699
Age, years	57.00 (50.00, 62.00)	56.00 (48.00, 62.00)	57.00 (49.00, 62.00)	57.00 (50.00, 63.00)	58.00 (51.00, 63.00)	58.00 (51.00, 63.00)	<0.001
Sex							<0.001
Women	95 295 (53.71)	15 722 (44.30)	18 173 (51.21)	19 187 (54.07)	20 278 (57.14)	21 935 (61.81)	
Men	82 146 (46.29)	19 767 (55.70)	17 315 (48.79)	16 301 (45.93)	15 210 (42.86)	13 553 (38.19)	
Townsend deprivation index	−2.40 (−3.77, −0.14)	−2.22 (−3.67, 0.23)	−2.39 (−3.75, −0.13)	−2.45 (−3.81, −0.26)	−2.49 (−3.82, −0.30)	−2.45 (−3.79, −0.22)	<0.001
Body mass index, kg/m^2^	26.25 (23.75, 29.27)	27.06 (24.47, 30.17)	26.50 (24.00, 29.51)	26.25 (23.78, 29.23)	25.99 (23.56, 28.92)	25.46 (23.12, 28.38)	<0.001
Energy intake, kcal/d	2008.03 (1681.76, 2379.72)	1900.94 (1543.78, 2310.36)	1975.29 (1641.66, 2356.77)	2012.64 (1696.55, 2375.53)	2043.75 (1731.94, 2397.24)	2087.28(1786.04, 2439.20)	<0.001
Education levels							<0.001
High	75 290 (42.43)	11 335 (31.94)	14 040 (39.56)	15 413 (43.43)	16 553 (46.64)	17 949 (50.58)	
Middle	86 391 (48.69)	19 493 (54.93)	18 001 (50.72)	17 096 (48.17)	16 402 (46.22)	15 399 (43.39)	
No above	15 088 (8.50)	4447 (12.53)	3292 (9.28)	2860 (8.06)	2434 (6.86)	2055 (5.79)	
Unknown/missing	672 (0.38)	214 (0.60)	155 (0.44)	119 (0.34)	99 (0.28)	85 (0.24)	
Annual household income							<0.001
<£18 000	23 845 (13.44)	5552 (15.64)	4976 (14.02)	4654 (13.11)	4287 (12.08)	4376 (12.33)	
£18 000–£29 999	38 427 (21.66)	7728 (21.78)	7641 (21.53)	7583 (21.37)	7710 (21.73)	7765 (21.88)	
£30 000–£51 999	45 894 (25.86)	8983 (25.31)	9259 (26.09)	9168 (25.83)	9226 (26.00)	9258 (26.09)	
£52 000–£100 000	39 942 (22.51)	7384 (20.81)	7891 (22.24)	8193 (23.09)	8301 (23.39)	8173 (23.03)	
>£100 000	11 770 (6.63)	1998 (5.63)	2191 (6.17)	2469 (6.96)	2548 (7.18)	2564 (7.22)	
Unknown/missing	17 563 (9.90)	3844 (10.83)	3530 (9.95)	3421 (9.64)	3416 (9.62)	3352 (9.45)	
Smoking status							<0.001
Current	13 599 (7.66)	4460 (12.57)	2905 (8.19)	2410 (6.79)	2141 (6.03)	1683 (4.74)	
Never	100 104 (56.42)	18 404 (51.86)	19 871 (55.99)	20 200 (56.92)	20 613 (58.08)	21 016 (59.22)	
Previous	63 372 (35.71)	12 527 (35.30)	12 609 (35.53)	12 829 (36.15)	12 677 (35.72)	12 730 (35.87)	
Unknown/missing	366 (0.21)	98 (0.28)	103 (0.29)	49 (0.14)	57 (0.16)	59 (0.17)	
Alcohol drinking status							<0.001
Current	167 694 (94.51)	33 395 (94.10)	33 520 (94.45)	33 645 (94.81)	33 578 (94.62)	33 556 (94.56)	
Never	4545 (2.56)	958 (2.70)	904 (2.55)	853 (2.40)	941 (2.65)	889 (2.50)	
Previous	5132 (2.89)	1114 (3.14)	1041 (2.94)	985 (2.78)	962 (2.71)	1030 (2.90)	
Unknown/missing	70 (0.04)	22 (0.06)	23 (0.06)	5 (0.01)	7 (0.02)	13 (0.04)	
Family history of cancer							<0.001
No	99 853 (56.28)	19 663 (55.41)	19 881 (56.02)	20 022 (56.42)	20 141 (56.75)	20 146 (56.77)	
Yes	62 729 (35.35)	12 437 (35.04)	12 541 (35.34)	12 487 (35.19)	12 561 (35.40)	12 703 (35.80)	
Unknown/missing	14 859 (8.37)	3389 (9.55)	3066 (8.64)	2979 (8.39)	2786 (7.85)	2639 (7.43)	
Dietary component intake, g/d							
Vegetables	284.75 (132.00, 476.62)	90.00 (0.00, 187.00)	208.81 (90.00, 353.00)	299.50 (173.00, 462.44)	383.75 (245.00, 556.75)	473.06 (324.00, 661.75)	<0.001
Fruits	280.00 (130.00, 460.00)	100.00 (0.00, 220.00)	220.00 (106.00, 372.00)	296.00 (170.00, 459.00)	352.00 (217.00, 527.00)	423.33 (279.00, 609.08)	<0.001
Legumes	32.50 (0.00, 70.00)	0.00 (0.00, 0.00)	0.00 (0.00, 35.00)	32.50 (0.00, 70.00)	65.00 (0.00, 118.75)	70.00 (65.00, 135.00)	<0.001
Nuts	0.00 (0.00, 30.00)	0.00 (0.00, 0.00)	0.00 (0.00, 0.00)	0.00 (0.00, 20.00)	10.00 (0.00, 40.00)	40.00 (20.00, 47.00)	<0.001
Fish	0.00 (0.00, 140.00)	0.00 (0.00, 0.00)	0.00 (0.00, 0.00)	0.00 (0.00, 120.00)	92.00 (0.00, 140.00)	140.00 (92.00, 190.00)	<0.001
Unsaturated fats	13.17 (9.26, 17.99)	10.82 (7.16, 15.94)	12.20 (8.46, 17.02)	13.04 (9.44, 17.69)	13.93 (10.27, 18.37)	15.43 (11.55, 20.21)	<0.001
Total grains	150.00 (44.00, 274.00)	44.00 (0.00, 180.00)	101.75 (38.00, 243.50)	159.00 (44.00, 280.00)	203.50 (80.00, 310.00)	224.00 (124.00, 305.00)	<0.001
Potatoes	175.00 (0.00, 223.75)	87.50 (0.00, 180.00)	175.00 (0.00, 180.00)	175.00 (0.00, 245.00)	175.00 (87.50, 310.00)	180.00 (90.00, 355.00)	<0.001
Eggs	0.00 (0.00, 50.00)	0.00 (0.00, 50.00)	0.00 (0.00, 50.00)	0.00 (0.00, 50.00)	0.00 (0.00, 66.67)	0.00 (0.00, 90.00)	<0.001
Poultry	0.00 (0.00, 130.00)	0.00 (0.00, 100.00)	0.00 (0.00, 130.00)	0.00 (0.00, 130.00)	0.00 (0.00, 130.00)	0.00 (0.00, 130.00)	<0.001
Dairy foods	95.00 (20.00, 165.00)	20.00 (0.00, 80.00)	55.00 (0.00, 145.00)	102.50 (20.00, 165.00)	125.00 (40.00, 180.00)	145.00 (83.33, 205.00)	<0.001
Red meat	92.00 (0.00, 158.00)	120.00 (23.00, 143.00)	98.00 (0.00, 150.00)	115.00 (0.00, 166.00)	113.00 (0.00, 180.00)	46.00 (0.00, 152.00)	<0.001
Saturated fats	28.20 (21.12, 36.66)	28.28 (20.58, 37.75)	28.14 (20.79, 36.79)	28.16 (21.10, 36.45)	28.05 (21.33, 36.19)	28.39 (21.77, 36.23)	0.003
Added sugar	0.00 (0.00, 4.25)	0.00 (0.00, 8.50)	0.00 (0.00, 4.25)	0.00 (0.00, 4.25)	0.00 (0.00, 4.25)	0.00 (0.00, 4.25)	<0.001

Continuous variables are presented as median (interquartile range), and categorical variables are presented as *n* (%).

Individuals ranked in the highest quintile of PHD scores tended to be older, female, better education levels, had greater total energy intake, lower BMI, lower Townsend deprivation index, were more likely to have never smoked, currently consume alcohol, and had no family history of cancer. Furthermore, they also reported higher consumption of vegetables, fruits, legumes, nuts, fish, unsaturated fats, total grains, potatoes, dairy, red meat, and saturated fats.

### Association of PHD with cancer incidence

**Figure 2.**
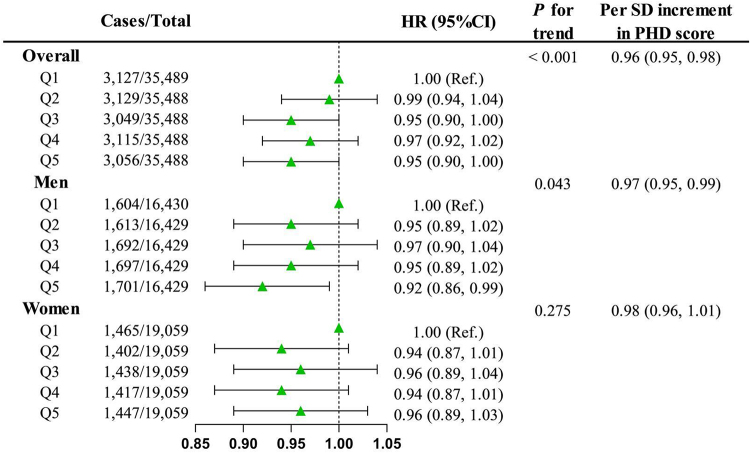
Associations between PHD score and overall cancer incidence.

CI, confidence interval; HR, hazard ratio; PHD, planetary health diet, Q, quintile; Ref, reference; SD, standard deviation.The Cox model was adjusted for age, body mass index, energy intake, the first ten genetic principal components, education levels, annual household income, Townsend deprivation index, alcohol drinking status, smoking status, and family history of cancer.

**Figure 3.**
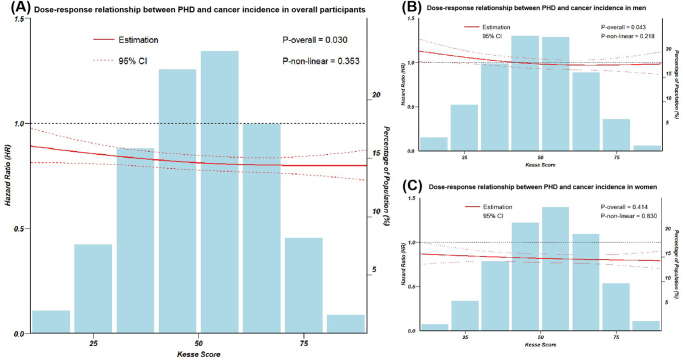
(A) Dose-response relationship between PHD and cancer incidence in overall participants. (B) Dose-response relationship between PHD and cancer incidence in men.

After adjusting for confounders, each SD increase in PHD score was associated with a 4% decreased risk of developing cancer (HR: 0.96, 95% CI: 0.95–0.98). Participants with the highest PHD score were associated with a lower risk of overall cancer (HR: 0.95, 95% CI: 0.90–1.00). The RCS analysis indicated a clear linear dose-response relationship (*P* for nonlinearity >0.05) (Figs. 2, 3).

(C) Dose-response relationship between PHD and cancer incidence in women. Adjusted for age, body mass index, energy intake, the first 10 genetic principal components, education levels, annual household income, Townsend deprivation index, alcohol consumption, smoking status, and family history of cancer.

For men, each SD increase in PHD score associated with a 3% lower cancer risk (HR: 0.97, 95% CI: 0.95–0.99). Compared with participants in the lowest PHD score quintile, those with the highest PHD score were associated with lower cancer risk (HR: 0.92, 95% CI: 0.86–0.99) in a linear relationship (*P* for nonlinearity >0.05) (Figs. 2, 3). Specifically, elevated PHD scores in men were linked with a decreased risk of colorectal cancer (HR: 0.84, 95% CI: 0.70–1.00), lung cancer (HR: 0.57, 95% CI: 0.42–0.76), and esophageal cancer (HR: 0.54, 95% CI: 0.35–0.82) (Supplemental Digital Content Figure 1, available at: http://links.lww.com/JS9/E414).

However, in women, no significant associations were observed for overall cancer or most types of cancer, except for lung cancer (HR: 0.60, 95% CI: 0.44–0.81) (Figs. 2, 3 and Supplemental Digital Content Figure 1, available at: http://links.lww.com/JS9/E414).

In the analysis of subgroups based on age, BMI, smoking, and alcohol consumption, the results were consistent with the main findings, although not all of them reached statistical significance (Supplemental Digital Content Figure 2, available at: http://links.lww.com/JS9/E414). In the sensitivity analyses, excluding participants who developed cancer within the first 2 years, or who had completed the dietary questionnaire only once or not at all, and reconstructing the PHD score, the major results were also generally robust (Supplemental Digital Content Tables 5–7, available at: http://links.lww.com/JS9/E414).

### Association of CPRS with cancer incidence

**Figure 4.**
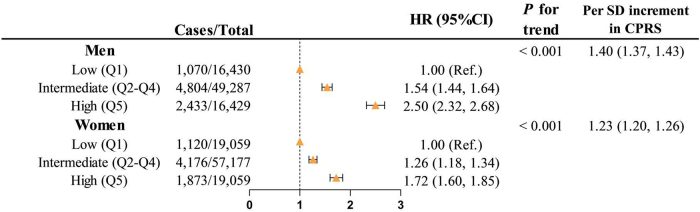
Associations between genetic risk and overall cancer incidence.

CI, confidence interval; CPRS, Cancer Polygenic Risk Scores; HR, hazard ratio; Q, quintile; Ref, reference; SD, standard deviation.The Cox model was adjusted for age, body mass index, energy intake, the first 10 genetic principal components, education levels, annual household income, Townsend deprivation index, alcohol drinking status, smoking status, and family history of cancer.

The associations between genetic risk and cancer incidence are presented in Figure 4. In the multivariable-adjusted model, each SD increase in CPRS was associated with a 40% and 23% elevation in overall cancer risk in men (95% CI: 1.37–1.43) and in women (95% CI: 1.20–1.26). A high CPRS was linked to an increased cancer risk. Compared with the low genetic risk group, multivariable-adjusted HRs for overall cancer risk in men were 1.54 (95% CI: 1.44–1.64) and 2.50 (95% CI: 2.32–2.68) in the intermediate and high genetic risk groups, while in women, they were 1.26 (95% CI: 1.18–1.34) and 1.72 (95% CI: 1.60–1.85), with all *P* for trend <0.001.

### Joint association of PHD and CPRS with cancer incidence

In the joint association analysis, the high CPRS and low PHD score group were used as reference. A significant reduction in cancer risk was observed among individuals with high PHD scores and low CPRS, in both men (HR: 0.39, 95% CI: 0.33–0.46) and women (HR: 0.55, 95% CI: 0.47–0.65) (Supplemental Digital Content Table 8, available at: http://links.lww.com/JS9/E414).

## Discussion

In this prospective cohort study based on UKB database, we found that higher adherence to the PHD was associated with a reduced cancer risk. When stratified by sex, this association was observed in men and was notably linked to particular cancers including colorectal, lung, and esophageal cancers, while a significant association was observed only for lung cancer in women. Individuals with high PHD scores and low CPRS had the relatively lowest risk of overall cancer, with the association being more pronounced in men. These findings highlight the role of sex and genetic factors in the relationship between PHD and cancer risk, suggesting that future research and intervention strategies should carefully consider these factors to optimize health management outcomes.

Dietary patterns are modifiable factors that influence cancer risk. Many studies have demonstrated a protective correlation between high-quality dietary habits, such as diets abundant in fruits, vegetables, whole grains, and lean proteins, and reduced cancer risk^[38–40]^. The PHD has attracted considerable attention for its focus on both improving human health and tackling global environmental sustainability issues. In our study, individuals with the highest adherence to the PHD exhibited lower overall cancer risk than those in the lowest quintile. Furthermore, the RCS analysis suggested a primarily linear association between PHD adherence and cancer risk. This may explain the slight inconsistency between the borderline significance observed in the highest quintile and the significant per-SD association, as categorization into quintiles can lead to information loss compared with continuous modeling. This finding aligns with a large cohort study suggesting that strict adherence to the PHD may reduce cancer risk by 39% over a 20-year follow-up[41]. However, the data based on the NutriNet-Santé cohort suggested that adherence to the PHD diet was not associated with cancer risk, in the multivariable model[10]. The discrepancy may stem from the sample size, as the study population and outcome events in the NutriNet-Santé cohort were less than half of those in the present study. Our research also confirmed that higher adherence to the PHD correlated with a decreased risk of specific cancers. In particular, we observed that higher adherence to the PHD correlated with a reduced risk of lung cancer, consistent with findings from another prospective study[42]. Although the methods for calculating PHD scores differed between studies, the consistency of results reinforces the robustness of our findings. Similarly, data from the multi-center prospective cohort study, the Prostate, Lung, Colorectal, and Ovarian trial, further confirmed the association between the PHD diet and lung cancer risk[43]. The reduction in lung cancer risk may be attributed to the bioactive components abundant in the PHD diet, such as flavonoids, which have antioxidant properties that scavenge free radicals and repair DNA damage^[44–47]^. Additionally, another cohort study showed an inverse correlation between the PHD diet and 12 inflammatory markers, most of which appeared to mediate the observed association[48], suggesting that inflammation regulation as a potential biological mechanism. These findings underscore the potential public health benefits of promoting PHD adherence as a modifiable dietary strategy for cancer prevention.

Another important finding was the significant sex difference in the protective role of the PHD, potentially attributed to factors such as hormonal differences and varying dietary habits between men and women. Compared with the lowest PHD quintile, a significant reduction in overall cancer risk was observed only among men with PHD scores in the highest quintile. Combined analysis revealed a stronger protective association in men than in women. This finding aligns with a recent research that revealed a significant sex difference, where the reduced cancer risk was observed only among men adhering to the PHD[11]. In addition, data from a multiethnic cohort study indicate a strong link between plant-based diets and a reduced colon cancer risk in men[49]. One potential explanation for this discrepancy is difference in sex hormone levels. Specifically, plant-based foods in the PHD, such as legumes and whole grains, are rich in phytochemicals like isoflavones, which can mitigate the cancer-promoting influence of androgens by downregulating androgen receptor expression[50]. This effect may further strengthen the protective association between the PHD diet and reduced cancer risk in men. Furthermore, in our study, among individuals over the age of 60 who experienced significant declines in hormone levels, following the PHD demonstrated similar protective associations in both men and women, further supporting this hypothesis. Another possible explanation may stem from variations in dietary habits between men and women. Research indicates that dietary patterns are strongly associated with gender^[51,52]^. Generally, men tend to prefer high-fat, high-calorie, animal-based diets[53]. However, men in the highest quintile of PHD adherence were more likely to adopt a plant-based diet, thereby reducing potential cancer-related harmful exposure to animal-derived proteins, such as heterocyclic amines^[54,55]^. In contrast, women are naturally more inclined toward plant-based diets^[56,57]^, which may explain why the cancer risk reduction was not as pronounced in women as in men. These findings provide valuable insights into the sex-specific effects of dietary patterns on cancer risk, highlighting the need for personalized dietary recommendations in cancer prevention strategies.

The interaction between genetic and environmental factors plays a crucial role in cancer development^[18,19]^. Environmental factors, particularly dietary patterns, can substantially impact cancer risk in individuals with genetic susceptibility. In our research, individuals with low genetic risk and great adherence to the PHD exhibited a relatively lowest cancer risk than those with high genetic risk and poor adherence to the diet. This phenomenon might be linked to the high levels of natural compounds in the PHD, such as resveratrol and flavonoids, which can regulate gene expression by means of epigenetic mechanisms, including DNA methylation and histone modification, thereby modifying genetic risk^[58,59]^. Epigenetic studies show that environmental factors, such as diet, can affect gene expression without changing the DNA sequence. In other words, diet may influence cancer risk by modulating genetic susceptibility through these epigenetic pathways^[60,61]^. Furthermore, antioxidants in the PHD, such as vitamin C and polyphenols, can neutralize free radicals and reduce oxidative stress, thereby lowering the risk of DNA damage^[62,63]^. Oxidative stress is widely recognized as a major factor in cancer development[64]. A previous study found that an antioxidant-rich diet reduced the risk of breast cancer in BRCA mutation carriers[65].

The primary advantages of this study are as follows. To our knowledge, this is the first prospective cohort study to evaluate sex-specific associations between the PHD, genetic risk, and cancer risk. In addition, the extensive sample size from the UKB, with over 177 000 participants, and the use of CPRS provided robust statistical power and a comprehensive assessment of genetic susceptibility. The prospective cohort design enabled long-term follow-up, further enhancing the reliability of our findings. Unlike previous studies where food intake was categorized based on specific thresholds[11], we employed a refined assessment method that categorized 14 dietary components into three groups based on their health impacts, using a flexible 0–10 scoring system to reflect intake levels and their nonlinear relationship with health outcomes[7], thereby enabling tailored dietary modifications. This approach allowed for precise measurement of adherence to the PHD and detailed assessment of dietary patterns. Despite the study by Karavasiloglou *et al*[11] revealed the role of dietary patterns in cancer risk, it did not fully account for individual differences in genetic background. Therefore, we introduced PRS to construct CPRS, enabling us to assess the impact of dietary patterns and individual genetic susceptibility on cancer risk^[16,17]^, providing insights for personalized health interventions across different genetic risk backgrounds.

few limitations exist in this study. First, the 24-hour online dietary questionnaire may not fully capture habitual eating patterns and is subject to recall bias, which could lead to misclassification and measurement inaccuracies[66]. To mitigate this, we excluded participants with only one dietary recall and used the average intake for those with multiple records. Additionally, the classification of foods into adequacy, optimum, and moderation categories with a scoring system aimed to better represent dietary variations and reduce potential bias^[7,30]^. However, residual dietary measurement errors cannot be entirely ruled out. Second, the study population predominantly consists of individuals of European descent and healthy volunteers. The relatively homogeneous genetic backgrounds and dietary habits in the UKB may restrict the applicability of our results to more diverse populations[67]. Future studies involving more diverse cohorts are needed to confirm the generalizability of these findings. Third, although we adjusted for multiple covariates, residual confounding remains a concern, particularly regarding unmeasured genetic and environmental factors that could influence dietary intake and health outcomes. Fourth, the non-significant association observed in the female subgroup may be influenced by limited statistical power due to a relatively small number of cancer cases, reducing the ability to detect modest associations. Future studies with larger female cohorts may help clarify whether the observed differences are due to true biological variations or sample size limitations. Fifth, the use of categorical analysis may have reduced statistical power and obscured dose-response trends that continuous models might capture, possibly explaining the borderline significance for the highest PHD quintile in the overall population. Lastly, as an observational study, our findings cannot establish causal relationships, emphasizing the need for future studies with more robust designs to validate our results.

## Conclusion

This large-scale prospective cohort study demonstrated that the PHD was associated with a reduced overall cancer risk in men. Individuals with high PHD scores and low CPRS had the relatively lowest cancer risk. These findings highlight the role of gender-specific differences in the association between diet and genetic factors, providing practical insights to optimize cancer prevention strategies. Future research should focus on longitudinal studies and intervention trials to further elucidate the causal mechanisms underlying these associations and assess the long-term effectiveness of dietary interventions in cancer prevention.

## Data Availability

UK Biobank data can be requested by researchers for approved projects, including replication, through https://www.ukbiobank.ac.uk/.

## Research registration unique identifying number (UIN)

This research has been conducted using the UK Biobank Resource under Application Number 81680. UK Biobank data have approval from the North West Multi-Centre Research Ethics Committee (MREC) (REC reference: 21/NW/0157).
